# Facing metal stress by multiple strategies: morphophysiological responses of cardoon (*Cynara cardunculus* L.) grown in hydroponics

**DOI:** 10.1007/s11356-021-13242-9

**Published:** 2021-03-14

**Authors:** Maria Cristina Sorrentino, Fiore Capozzi, Chiara Amitrano, Gaetano De Tommaso, Carmen Arena, Mauro Iuliano, Simonetta Giordano, Valeria Spagnuolo

**Affiliations:** 1 Dipartimento di Biologia, Università degli Studi di Napoli Federico II, Complesso Universitario di Monte Sant’Angelo, Cupa Nuova Cintia, 21-80126 Napoli, Italy; 2 Dipartimento di Agraria, Università degli Studi di Napoli Federico II, Via Università, 100-80055 Portici, Italy; 3 Dipartimento di Scienze Chimiche, Università degli Studi di Napoli Federico II, Complesso Universitario di Monte Sant’Angelo, Cupa Nuova Cintia, 21-80126 Napoli, Italy

**Keywords:** Cardoon, Stomata, Root hairs, Gas exchanges, Cadmium, Lead

## Abstract

The contamination of environments by heavy metals has become an urgent issue causing undesirable accumulations and severe damages to agricultural crops, especially cadmium and lead which are among the most widespread and dangerous metal pollutants worldwide. The selection of proper species is a crucial step in many plant-based restoration approaches; therefore, the aim of the present work was to check for early morphophysiological responsive traits in three cultivars of *Cynara cardunculus* (Sardo, Siciliano, and Spagnolo), helping to select the best performing cultivar for phytoremediation. For all three tested cultivars, our results indicate that cardoon displays some morphophysiological traits to face Cd and Pb pollution, particularly at the root morphology level, element uptake ability, and photosynthetic pigment content. Other traits show instead a cultivar-specific behavior; in fact, stomata plasticity, photosynthetic pattern, and antioxidant power provide different responses, but only Spagnolo cv. achieves a successful strategy attaining a real resilience to metal stress. The capacity of Spagnolo plants to modify leaf structural and physiological traits under heavy metal contamination to maintain high photosynthetic efficiency should be considered an elective trait for its use in contaminated environments.

**Supplementary Information:**

The online version contains supplementary material available at 10.1007/s11356-021-13242-9.

## Introduction

Rapid urbanization and agricultural practices can be considered the main responsible for metal pollution of land, water, and plants, with a generally detrimental effect for all living organisms. Although heavy metals are natural components of soil at trace levels, and some of them (i.e., Cu and Zn) are essential for normal plant growth and development, the increasing anthropogenic activities have contributed to their accumulations. Urbanization involves industrialization and massive vehicular traffic, which release pollutants to air, soil, and water. Moreover, farmland activities sometimes imply the use of contaminated irrigation water, and mineral fertilizers, herbicides, and additives for animal feeds, with a global increase in metal pollution. As a consequence of current land-use practices, or activities inducing heavy metal mobilization, many soils are contaminated by these pollutants, especially in the form of pesticides and nutrients (Motuzova et al. 2014). In recent time, soil contamination by heavy metals has become an urgent issue, since it results in a severe loss in agricultural yield and damages in human health, due to metal entrance into the food web and biomagnification of some metals, such as Pb and Cd (He et al. 2015; Su et al. 2014).

The toxicity of heavy metals on plant metabolism has received extensive interest for several decades (Markert 1993), with particular attention to crops (Chaney 2015), whose contamination can cause health problems, as these plants are directly related to the human diet. Cadmium (Cd) and lead (Pb) are among the most widespread metal pollutants worldwide (Su et al. 2014). Pb naturally occurs in soils, but it is present in relatively low concentrations. In uncontaminated soils, Pb is generally in the range of 20-50 mg kg^-1^ (Nriagu 1978). Nonpolluted soils usually contain less than 100 mg kg^-1^ Pb; soils in unpolluted polar areas, buried before the industrial revolution, contain less than 5 mg kg^-1^ (Meggeson and Hall 1999). In industrialized areas, up to 1000 mg kg^-1^ Pb and above has been recorded (Angelone and Bini 1992; Capozzi et al. 2020). In recent studies carried out in Southern Italy, aimed at the environmental restoration of metal-contaminated areas, some industrial sites were selected, with Cd and Pb concentrations ranging between 1.6 and 314 mg kg^-1^, and 409 and 100,000 mg kg^-1^, respectively (Capozzi et al. 2020; Visconti et al. 2018).

Cadmium naturally occurs in Zn, Pb, and Cu ores with Zn concentration exceeding 50-200-folds Cd; the mining and smelting of these ores involve Cd contamination of surrounding soils (Chaney 2015).

In plants, the exposition to heavy metals, and especially Cd, results in a severe reduction of plant growth and development as a consequence of photosynthetic apparatus impairment (Arena et al. 2014). Photosynthesis is one of the processes sensitive to metal toxicity (Arena et al. 2017a, b; Figlioli et al. 2019; Krupa and Baszyński 1995; Sorrentino et al. 2018). Metals may have multidirectional effects on photosynthesis (for a review see Prasad and Strzalka 1999). The excess of Cd or Pb, for example, affects the photosynthetic electron transport (Krupa and Baszyński 1995; Myśliwa-Kurdziel et al. 2002), as well as the activities of Calvin-Benson cycle enzymes, or the net assimilation of CO_2_ (Arena et al. 2017a, b; Prasad and Strzalka 1999; Sorrentino et al. 2018). Photosynthetic activity can also be altered indirectly by heavy metals, for example, decreasing the content of photosynthetic pigments, altering chloroplast ultrastructure (Molas 2002; Sorrentino et al. 2018), and lipid and protein composition of thylakoids (Skórzyńska-Polit and Baszyński 1997). Cadmium and Pb accumulation in leaves interferes with the stomata functioning, affecting the overall photosynthesis and transpiration rates (Chandra and Kang 2016). The plant physiological response against heavy metals is strictly depending on the species, being linked to the plant individual capability to face stress. Some species are powerful bioaccumulators (Arena et al. 2017a, b) and used in phytoremediation of contaminated soils.

In previous works (Capozzi et al. 2020; Sorrentino et al. 2018) carried out on *Cynara cardunculus* L. var. altilis DC., three cultivars were grown on a gardening soil and on industrial soil containing Cd and Pb, and different behaviors were observed under metal stress compared to the control plants. Although all cultivars could uptake significant amounts of metals, only *C. cardunculus* cv Spagnolo counteracted metal stress preserving chloroplast ultrastructure and increasing photosynthetic efficacy. By contrast, in the other cultivars analyzed, Cd and Pb uptake was coupled to the decrease in life span, pigment content, and photosynthetic activity. The selection of a species for phytoremediation of metal-polluted soils involves a deep knowledge of the mechanisms at the basis of plant resistance against metal stress; based on previous experimental evidences, *C. cardunculus*, cv Spagnolo, seems to be an eligible candidate for phytoremediation. We hypothesized that Cd and Pb, provided in hydroponic cultures, could affect other morphophysiological traits, useful as an early proxy of metal stress in the selection of phytoremediation species. Therefore, the aim of the present work was to verify if cultivar-specific responses occurred, considering (i) the number of stomata and the total stomata surface in relation to gas exchanges, (ii) root morphology in relation to metal uptake and translocation, and (iii) pigment content, photochemical efficiency, and antioxidant response.

## Materials and methods

### Plant material and growth conditions

The seeds of three cultivars of *Cynara cardunculus* var. *altilis* DC. (provided by Arca 2010 scarl), named Sardo (SAR), Siciliano (SIC), and Spagnolo (SPA), were germinated on wet filter paper for five days in the dark. Once primary roots and cotyledons were fully developed, 15 seedlings of each cultivar were moved to a hydroponic floating system, consisting of polystyrene plug trays floating in plastic tanks containing a constant volume of 5 L of aerated nutrient solution (Hoagland and Arnon 1950) at pH 5.5. Three tanks were used for the experimental plan: 5 seedlings of each cultivar per tank were exposed to a specific heavy metal or none as the control; at the two-true-leaf stage, CdCl_2_ and Pb(NO_3_)_2_ at the concentration of 10^-5^ M were supplied to the culture medium. The nutrient solution was renewed twice a week for 30 d. The parameters of the growth chamber were constantly monitored to maintain controlled conditions of temperature 24/18 °C, relative humidity (RH) 55-75% (day/night), and a photoperiod of 16 h light per day with a photosynthetic photon flux density (PPFD) at the top of the canopy of 180–200 μmol photons m^−2^ s^−1^. After 30 d culture, all plants were analyzed.

### Morphological traits of roots and stomata

**Fig. 1:**
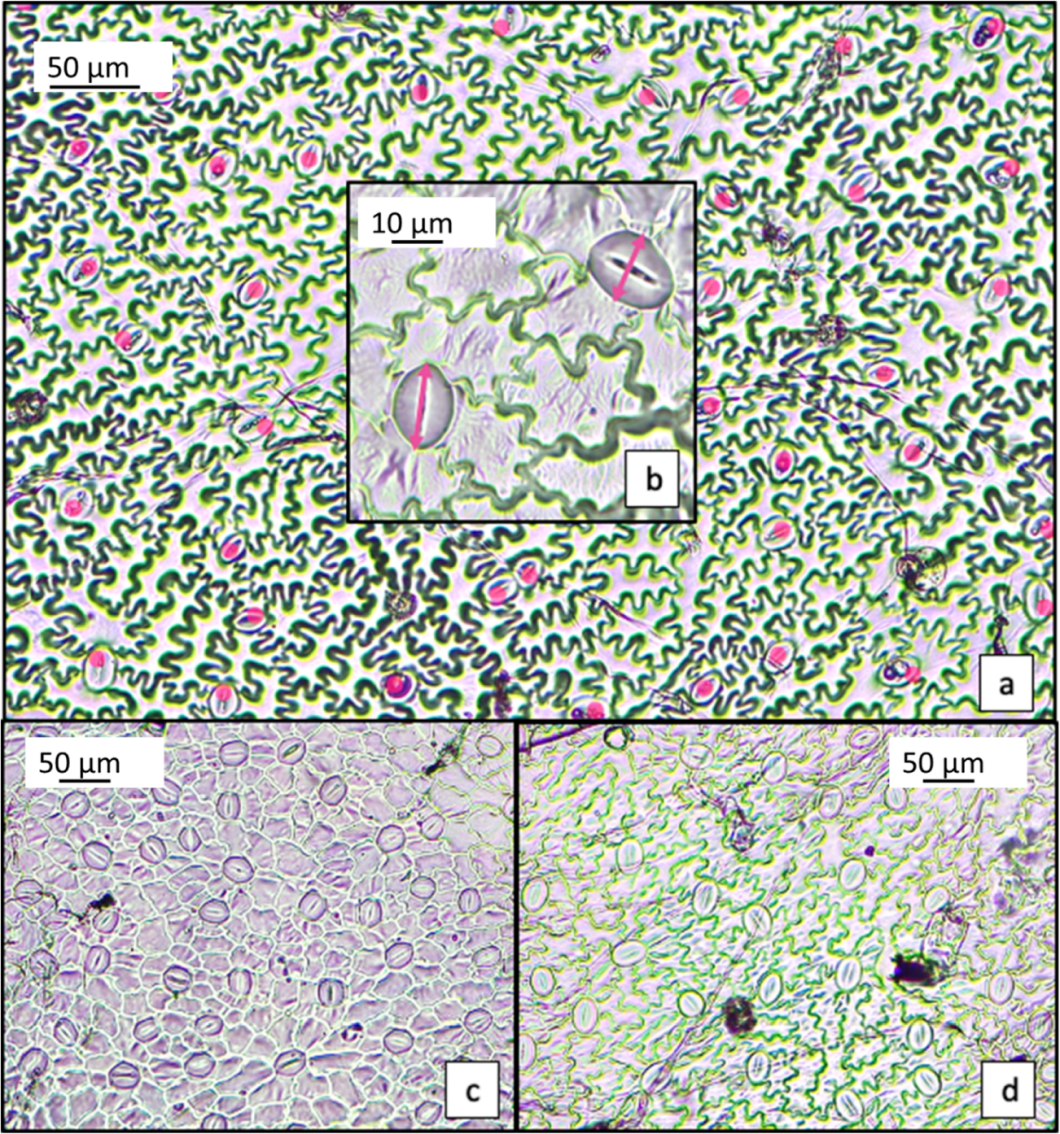
Example of stomata counting (a) and measuring (b) in a control sample of Spagnolo cardoon. Pb-treated samples of Spagnolo (c) and Siciliano (d) leaf replicas. Note the higher number of smaller stomata in the first; see paragraph 3.1 for further details

Root tips (5 for each thesis) from controls and metal-treated plants, at the end of the treatment, were cut under a stereomicroscope and observed under LM (Leica DME ICC50W) to estimate the presence of root hair and the extension of the hair zone. To determine the stomata size and number, four leaves of each cultivar were collected after the heavy metal exposure. All selected leaves were at the same age and development degree. A gentle peeling of the abaxial surface was carried out with scotch tape to remove trichomes. At the midlamina, four surface replicas were obtained by nail topcoat. For each thesis, 16 replicas were observed under a light microscope (*n* = 16, total analyzed surface = 0.8 mm^2^ × 16), and images acquired were analyzed by ImageJ software (National Institutes of Health, MD, USA) (Fig. 1a). Moreover, 20 stomata (i.e., 20 couples of guard cells) were measured for each thesis: both major and minor axes of each stoma were used to calculate its area as an ellipse (Fig. 1b). Then, this area was multiplied per the average stomata number found on a 0.8 mm^2^ area, and this value was assumed as the total stomata surface (TSS).

### Chemical analysis and sequential elution technique

To evaluate the total amounts of intra- and extracellular fractions of Cd and Pb, a sequential elution technique was used. Treated samples were divided into two batches: one batch was directly analyzed for the total element content, after dehydration and acid digestion. The second batch was put in 20 mL of 20 mM Na_2_EDTA solution for 20 min and then rinsed in deionized water, to remove the extracellular soluble fraction of Cd or Pb, also including that bound to the cell wall (Branquinho and Brown 1994; Branquinho et al. 1997; Spagnuolo et al. 2011). Specifically, all samples (shoot and root for total element content and only shoot for sequential elution; *n*=5) were weighted and dried in an oven at 40 °C until constant weight. Dehydrated plant materials were ground in an agate mortar for the digestion phase. Samples represented by root or leaf powder of weight between 100 and 250 mg were digested in 5 mL HNO_3_ 65% (hyperpure, Carlo Erba) and 2.5 mL H_2_O_2_ 30% (Sigma Aldrich) for metal analysis.

The digested samples were diluted to 25 mL in Millipore water and subsequently filtered to analyze Pb and Cd by Flame AAS (Varian Spectra AA 220 FS). All Pb and Cd standard solutions, for calibration curves, were prepared in 0.1 M HNO_3_ by dilution of Pb(NO_3_)_2_ and Cd(NO_3_)_2_ stock solutions, respectively. Lead (II) and cadmium (II) nitrate stock solutions were prepared by dissolving lead or cadmium metal, 5N8 (Metal Research) with a nitric acid stock solution. The exact metal concentration, in both solutions, was determined by complexometric titration with EDTA. The reference plant material CTA-OTL-1 (oriental tobacco leaves) was also acid-digested and analyzed for Cd and Pb concentrations, to determine the recovery percentages of the two elements; they were 92 and 103%, respectively.

### Gas exchange analysis

For the gas exchange and fluorescence measurements, fully expanded leaves were collected at 30 d after sowing.

Leaf gas exchanges were measured with a modular gas exchange measuring system equipped with an infrared gas analyzer Qubit Systems S151 IRGA CO_2_ analyzer (Qubit Systems Inc., Kingston, Ontario, Canada) to measure, at different times, the concentration of CO_2_ in gas entering a leaf chamber, and the concentration of CO_2_ in the same gas after it leaves the chamber. The difference between influx and efflux CO_2_ concentrations allowed the calculation of the photosynthetic CO_2_ fixation rate. The system included a humidity/temperature sensor (Q-S161) which measured relative humidity of the air before and after it has passed through the leaf chamber plus temperature at the RH sensor. The RH differential between influx and efflux gas, the temperature, and the flow rate through the leaf chamber allowed calculation of leaf transpiration rates.

The youngest full expanded leaves (5 leaves per plant for each treatment) were chosen for the measurements. Each leaf was enclosed in the leaf chamber for about 5-8 minutes to allow the photosynthesis to reach the steady state. The environmental parameter fixed in the chamber was photosynthetic photon flux density (PPFD) of 800 μmol photons m^-2^ s^-1^, temperature of 25 °C±1, ambient CO_2_ concentration of 360 μmol CO2 mol^-1^, and RH 50%. The PPFD on cuvette was provided by a LED external source (A113, Qubit System) which supplies photosynthetically active radiation to the leaf with minimum heat load. Net photosynthesis (*A*_*N*_), transpiration (*E*), and stomatal conductance to vapor (*g*_*s*_) were calculated according to the LoggerPro software (Qubit Systems Inc., Canada) as reported in Sengupta et al. 2019.

Using the differential CO_2_ concentration, which is the difference between the influx and efflux CO_2_ (dif. CO_2_), following the molecular flow (MF) rate, net-photosynthesis was calculated as follows:

1 $$ {A}_N=\mathrm{dif}.{\mathrm{CO}}_2\times \mathrm{MF}\ \mathrm{transpiration}\ \mathrm{rate} $$

where MF = flow rate/[22.4 × (273 + Tair)/273]/60 × 10,000/ leaf area.

Transpiration (*E*) was also calculated automatically by the software by using the values of reference and analytical relative humidity (RH) values. Stomatal conductance was calculated as reported:

2 $$ {g}_s=E/\left(\mathrm{ws}-\mathrm{Anal}.\mathrm{w}\right) $$

where ws is the water vapor concentration in saturated air and Anal. w is the analyzed water vapor.

### Photosynthetic pigment content

Total chlorophylls and carotenoids were determined at 30 DAS on the same 5 leaves per treatment utilized for gas exchange and fluorescence emission measurements, according to Lichtenthaler (1987). Pigments were extracted from leaf disks of 0.2 cm^2^ by mortar and pestle in ice-cold 100% acetone and centrifuged at 5000 rpm for 5 min (Labofuge GL, Heraeus Sepatech, Hanau, Germany). The absorbance of supernatants was quantified by a spectrophotometer (Cary 100 UV-VIS, Agilent Technologies, Santa Clara, CA, USA) at 470, 645, and 662 nm and pigment concentration expressed in μg cm^-1^.

### Fluorescence emission measurements

Fluorescence emission measurements were also performed on five replicates per each treatment, coming from five different plants. A portable FluorPen FP100max fluorometer, equipped with a light sensor (Photon System Instruments, Brno, Czech), was used for measurements, following the procedure reported in Arena et al. (2017a). The ground fluorescence signal, *F*_*o*_, was induced on 30′ dark-adapted leaves, by a blue LED internal light of about 1–2 μmol m^−2^ s^−1^. The maximal fluorescence level in the dark, *F*_*m*_, was induced by a 1 s saturating light pulse of 3000 μmol m^−2^ s^−1^. The maximum quantum efficiency of PSII photochemistry, *F*_*v*_*/F*_*m*_, was calculated as (*F*_*m*_ − *F*_*o*_)/*F*_*m*_, according to Kitajima and Butler (1975). The fluorescence measurements in the light were performed utilizing an open leaf-clip suitable for measurements under ambient light. The quantum yield of PSII electron transport (Φ_PSII_) was determined according to Genty et al. (1989). The linear electron transport rate (ETR) was expressed following Krall and Edwards (1992), whereas the nonphotochemical quenching (NPQ) was calculated as described in Bilger and Björkman (1990).

### Antioxidant capacity determination

The antioxidant analysis was carried out following the procedure reported in Costanzo et al. 2020, by the ferric reducing antioxidant power assay (FRAP). More specifically, 0.25 g of powdered sample was mixed with 60:40 (v/v) methanol/water solution and centrifuged at 14000 rpm for 15 min at 4 °C. Then, an acetate buffer (1:16 300 mM pH 3.6) containing a mix of tripyridyltriazine (TPTZ) (1:1.6 10 mM TPTZ in 40 mM HCl) and FeCl_3_ (1:16 12 mM FeCl_3_) was added to the extracts. The absorbance was measured at 593 nm with a spectrophotometer (UV-VIS Cary 100, Agilent Technologies, Palo Alto, CA, USA) after 1 h of incubation at 4 °C, using Trolox (6-hydroxy-2,5,7,8-tetramethylchroman-2-carboxylic acid) as standard. The antioxidant capacity was expressed as μmol Trolox equivalents for mg of sample fresh weight (FW).

### Statistical analysis

Basic statistics for morphological parameters: number of stomata, the stomata area (μm^2^), and the total stomata surface (mean number of stomata found on a surface of 0.8 mm^2^ × mean stomata area, μm^2^) were calculated and performed in Excel.

Statistically significant differences among treatments were analyzed by one-way analysis of variance (ANOVA). Shapiro-Wilk and Kolmogorov-Smirnov tests were performed to check for normality. The Holm-Sidak and Tukey’s tests were applied for all multiple comparison procedures based on a significance level of *p*<0.05, for physiological and morphological parameters, respectively. The package Sigma-Plot 11.0 (Jandel Scientific, San Rafael, CA, USA) was used for statistical data processing.

## Results and discussion

### Responses on stomata

Results of stomata counting and stomata area calculation are reported in Table 1 and Fig. 1. The stomata number varied significantly depending on cultivar: control plants of Sardo cv. had the highest number of stomata for each surface unit (on average 213), almost twice the number of stomata counted in Spagnolo (on average 117, Fig. 1c). In both cardoon Sardo and Siciliano, metal treatments significantly decreased the stomata number. Moreover, alterations in stomata shape were occasionally observed in the latter (Fig. 1s—supplementary). A decrease in the stomata number was already observed in *Beta vulgaris* in response to an excess of the micronutrient Zn (Sagardoy et al. 2010).

**Table 1:** Stomata number and area in control and metal-treated cultivars of *C. cardunculus*

Cv	Treatment	Stomata number^§^ (N.)	Stomata area (μm^2^)	Total stomata surface^§^ (μm^2^)
		Mean±SD	Mean±SD	Mean±SD
Sardo	^*^C	213±19^ab^	383±50^c^	79921±13794^ab^
	Cd	152±28^cd^	531±84^ab^	51177±14022^b^
	Pb	145±67^cd^	340±73^c^	80541±37958^ab^
Siciliano	C	170±33^bc^	466±125^ab^	80213±29975^ab^
	Cd	107±18^d^	506±127^ab^	62809±18004^ab^
	Pb	100±7^d^	583±96^a^	47056±6268^b^
Spagnolo	C	117±7^d^	463±86^abc^	54372±12585^b^
	Cd	231±42^a^	447±46^bc^	92265±25328^a^
	Pb	168±51^bc^	385±60^c^	71502±26932^ab^

^*^C control, *SD* standard deviation. Different letters indicate significant differences (*p* < 0.05) among the treatments according to Tukey’s post hoc test. ^§^All parameters were estimated on a surface of 0.8 mm^2^

By contrast, in Spagnolo cv. grown under metal stress, the number of stomata significantly increased, especially with Cd, where stomata nearly doubled those of control plants. In addition, in Sardo and Siciliano cultivars, a noticeable increase of the stomata area was also observed (significant in Sardo treated with Cd). This result can be interpreted as a compensative response of the plants under metal stress, to counteract the decrease of the stomata number.

However, this response seems not enough to cope with the reduced number of stomata; in fact, only in the case of Pb-treated plants of Sardo, a complete recovery of the total stomata surface was achieved. In Spagnolo cardoon, the sole increase of the stomata number produced a parallel increase of the total stomata surface, with positive effect on gas exchange.

An increase of the stomata number in plants grown under the metal presence was already observed in the literature; in wheat seedlings grown with Cu and Zn, the number of stomata increased with a dose-dependent trend (Stolarska et al. 2007). In soybean seedlings grown in the presence of Pb, an increase of the stomata number coupled to a reduction in their size was observed (Weryszko-Chmielewska and Chwil 2005). In the latter case, the authors suggested that a larger number of epidermal cells enhance detoxification mechanisms. Given that several papers report the negative effects of heavy metals on plant metabolic pathways, stomata which are more numerous but reduced in size could provide a prompt response in gas exchange. This could explain why in cardoon, where stomata undergo a double regulation pattern under metal stress (i.e., in number and size), the stomata number is more important than size for gas exchanges and photosynthesis.

### Responses on roots: morphology, element uptake, and translocation

**Fig. 2:**
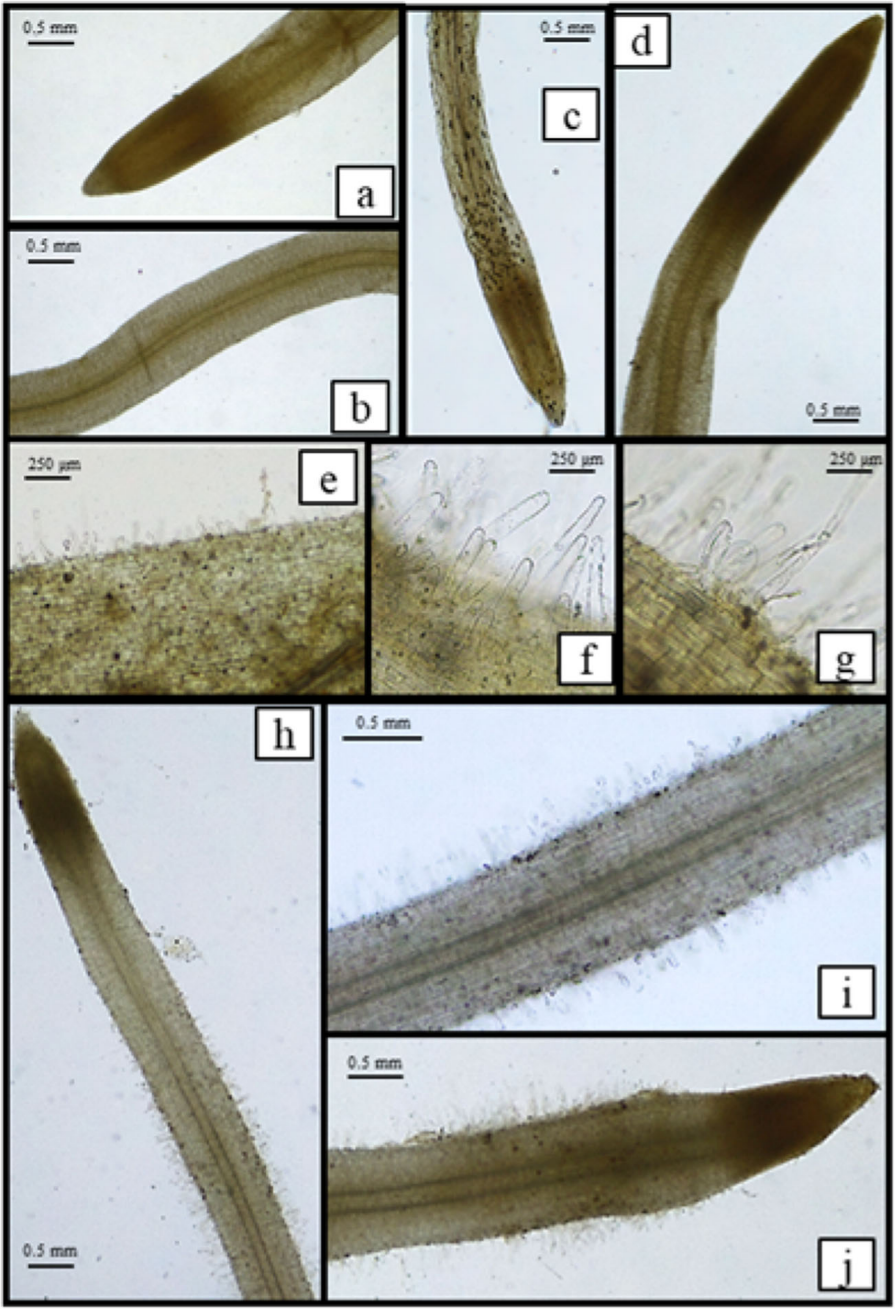
Roots of control plants of Spagnolo (a, b), Siciliano (c), and Sardo (d) cardoon cultivars. Roots of Cd-treated plants of Sardo, Siciliano, and Spagnolo (e, f, and g, respectively). Roots of Pb-treated plants of Sardo, Siciliano, and Spagnolo (h–j). See paragraph 3.2 for details

The observation of root apparatus after 30 d of culture (Fig. 2) highlighted the presence of 3-6 roots, similarly developed in control and metal-treated plants; however, the observation of the root tip under a light microscope evidenced the absence of root hairs in control plants, and the development of numerous root hairs in metal-treated plants, especially with Pb, where the hairy zone was about twice that of Cd-treated plants (7–8 mm vs. 3–4). The presence of root hairs could be interpreted as a plant response to metal stress, overall enhancing the root surface, cell wall binding sites for cations, and likely element absorption/adsorption and sequestration in the walls (Krzesłowska 2011; Parrotta et al. 2015).

Cadmium and Pb concentrations and their translocation factors (TF) in plants are reported in Table 2. Element concentrations were below the detection limit (BDL) in control plants. Significant differences were observed between different organs (i.e., root vs. leaves) and between EDTA-treated and untreated leaves. The EDTA washing demonstrates that a conspicuous part of Cd and Pb does not belong to the extracellular soluble fraction (removed by EDTA) but is likely located inside the cells. This could explain the noticeable physiological and ultrastructural alterations observed in a previous work in which cardoon plants were cultured under metal stress in industrial soil (Sorrentino et al. 2018). By contrast, no significant difference was observed between cultivars; these results agree with previous findings, showing that the same three cultivars did not display significant differences in their element uptake when grown on industrial soil (Capozzi et al. 2020). Moreover, in the present experiment, Cd and Pb concentrations in shoot and root resulted higher than those measured in a previous experiment carried out on gardening soil watered with metal solutions (Arena et al. 2017a, b), at parity of metal concentration (10^-5^ M) and growth period (30 d). This result is consistent in the two cases reflecting the amounts of metals received; in fact, the plants grown in gardening soil received about 600 mL metal solution each, whereas in hydroponics, every plant received about 1.5 L metal solution (10^-5^ M) in 30 d. In addition, the metals in solution could become partly inaccessible when supplied to the soil by irrigation; soil is indeed a complex matrix able to bind temporarily or stably metal ions, whereas they could preserve their bioavailability in hydroponics. Further, comparing the two experiments (gardening soil vs. hydroponics), in the first, translocation factors of 199% and 74% were calculated for Cd and Pb, values far long higher than those calculated in hydroponics (26–29% for Cd and 5–6% for Pb). All the same, it confirmed the higher value of TF for Cd. It is reported, indeed, that plant nutrients and Cd compete for the same transporters; therefore, Cd can be easily absorbed and translocated to the shoot (Nazar et al. 2012). This discrepancy could be due to the presence of root hairs, which develop only in metal-treated plants grown in hydroponics; root hairs could indeed provide a large surface for metal sequestration. The lack of root hairs in hydroponic control can be considered as an expected trait; in fact, root hairs develop to increase the root surface ensuring an adequate water supply, even in very small spaces, as between soil particles. The development of root hairs in Cd- and Pb-contaminated hydroponics could be a response to metal stress and especially to Pb, since the hairy zone was particularly developed in these plants and was coupled to a very low TF to the shoot.

**Table 2:** Element concentrations (ppm on dry weight, mean±SD, *n*=5) in leaf (L and L+EDTA), root (R), and relative translocation factor (TF) in the three cardoon cultivars

	Sardo	Siciliano	Spagnolo
Cd			
L	183±17^b^	152±19^b^	184±81^b^
L+EDTA	138±10^c^	102±14^c^	146±62^c^
R	693±53^a^	573±59^a^	642±75^a^
TF	0.26	0.26	0.29
Pb			
L	63±13^b^	48±11^b^	57±8^b^
L+EDTA	47±14^c^	29±6^c^	31±12^c^
R	824±102^a^	891±31^a^	1110±182^a^
TF	0.06	0.05	0.05

Different letters indicate statistical differences according to Tukey’s post hoc test (*p*<0.05)

### Gas exchanges

**Fig. 3:**
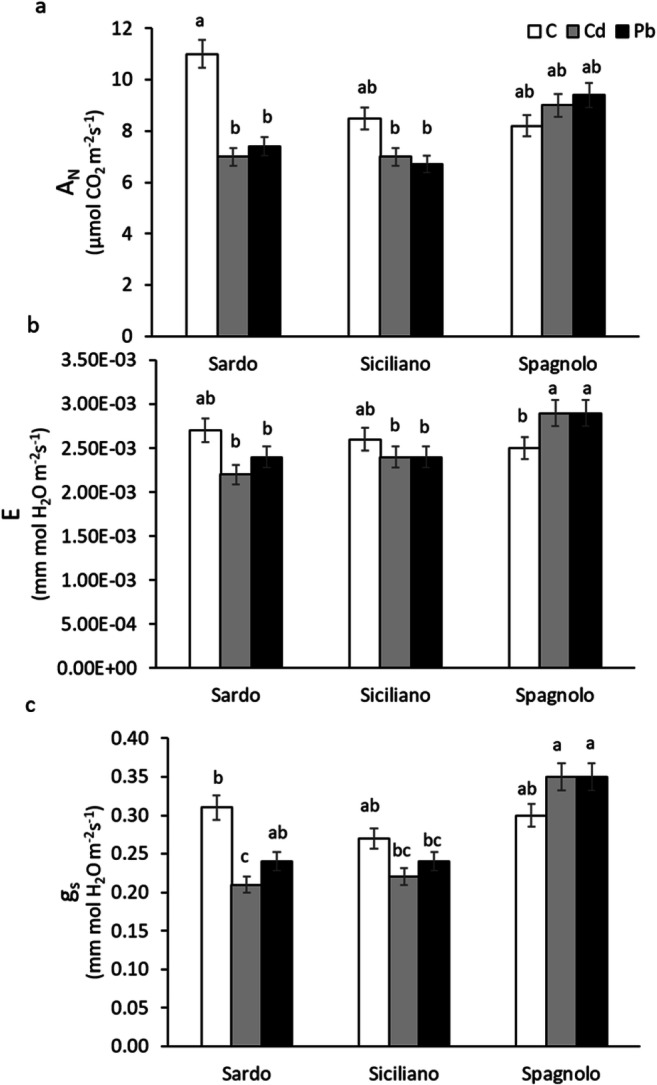
Net photosynthesis (*A*_*N*_) (a), transpiration rate (*E*) (b), and stomatal conductance (*g*_*s*_) (c) measured on control (C), Cd-treated, and Pb-treated Sardo, Siciliano, and Spagnolo cultivar. Data are the mean±SD, *n*=5. Different letters indicate significant differences among the treatments, according to Tukey’s post hoc test (*p*<0.05)

Leaf gas-exchange measurements are reported in Fig. 3. It is evident how Sardo and Siciliano cardoon showed the same trend of response in the measurements, being higher in control plants and lower in Cd and Pb, whereas Spagnolo showed a different physiological behavior, with no difference in net photosynthesis among control and treated plants (Fig. 3a) and a significant increase of transpiration and stomatal conductance in polluted plants (Fig. 3b, c). More specifically, Sardo presented the highest net photosynthesis and stomata conductance in nonpolluted plants, values which significantly decreased in Cd- and Pb-contaminated plants (Fig. 3a, b). These data were in agreement with those of the stomatal counting. Indeed, the highest presence of stomata could allow a higher stomata conductance, which in turn accounted for enhanced photosynthesis. Conversely, in Spagnolo grown under metal stress, there was an increase in both transpiration and stomatal conductance compared to control (Fig. 3b, c). Once again, these data agree with stomatal counting, where Spagnolo grown in the presence of Pb and Cd increased the stomata number. Indeed, gas exchanges in leaves are recognized to be controlled by several anatomical traits (i.e., stomatal number, size, and aperture), affecting the stomatal conductance and the diffusion through the mesophyll (Haworth et al. 2016; Xiong et al. 2017). From our results, it is also worth mentioning that Cd reduced stomata conductance and evapotranspiration more than Pb in the cultivars Sardo and Siciliano (Fig. 3).

### Photosynthetic pigment content

**Fig. 4:**
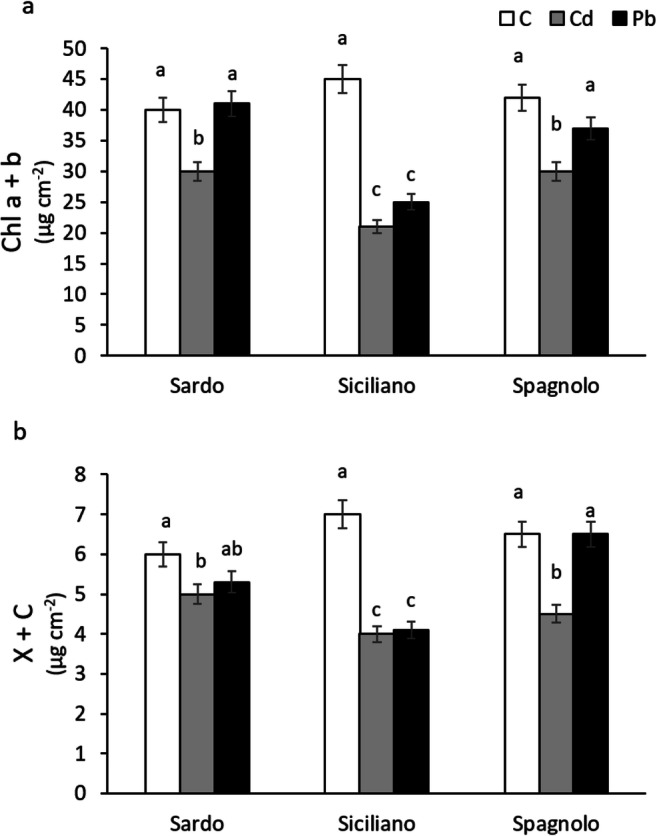
Total chlorophylls (Chl a+b) (a), total carotenoids (x+c) (b) determined on control (C), Cd-treated, and Pb-treated Sardo, Siciliano, and Spagnolo cultivar. Data are the mean±SD, *n*=5. Different letters indicate significant differences among the treatments, according to Tukey’s post hoc test (*p*<0.05)

Photosynthetic pigment content represents a suitable proxy for the detection of damages to photosynthetic apparatus. In our experiment, Spagnolo and Sardo showed a higher content of photosynthetic pigments (Fig. 4) in control and Pb plants, differently from Siciliano cultivar in which the pigment amount was significantly reduced under Cd and Pb treatments compared to control.

These results indicate a higher sensitivity to Cd, compared to Pb, in Spagnolo and Sardo cardoon, and an overall vulnerability of Siciliano cultivar to both metals regards pigments. The reduction of chlorophylls and carotenoids due to heavy metal contamination has often been reported as a damage occurring at the enzymatic level during the chlorophyll synthesis or by inducing oxidative stress that results harmful for the biosynthetic pathways of photosynthetic pigments (Prajapati and Tripathi 2008; De Micco et al. 2019). One of the most common results of injuries by heavy metal contamination is chlorosis. Metals interfere with pigment and influence the Chl content in plants as Mg^2+^ in chlorophyll is substituted by Cu^2+^, Zn^2+^, Cd^2+^, Hg^2+^, Pb^2+^, or Ni^2+^ (Kűpper et al. 1996). In our study, pigment content was significatively affected by heavy metals in Siciliano plants (Fig. 4) with repercussion on net photosynthesis (Fig. 3a).

Various authors have reported similar decreases in pigment content under heavy metal stress (Zengin and Munzuroglu 2006; Arena et al. 2014), whereas only a few have found enhanced concentrations of photosynthetic pigments after the exposure to metals (Devi Prasad and Devi Prasad 1982). These contradictory results are probably due to the effects of mixed heavy metals on the soil water medium, which compete with each other. Furthermore, in Sardo and Spagnolo, both chlorophyll and carotenoids decreased when treated with Cd but not with Pb. A significant inhibitory effect on photosynthetic pigments was found in our previous study with cardoon plants grown on polluted soil and was ascribed to the major presence of Cd (Sorrentino et al. 2018). This result confirms that cadmium is harmful to pigments probably because of its fast delivery through plants and accumulation in leaves compared to other heavy metals (Nwosu et al. 1995; John et al. 2009).

### Fluorescence emission measurements

**Fig. 5:**
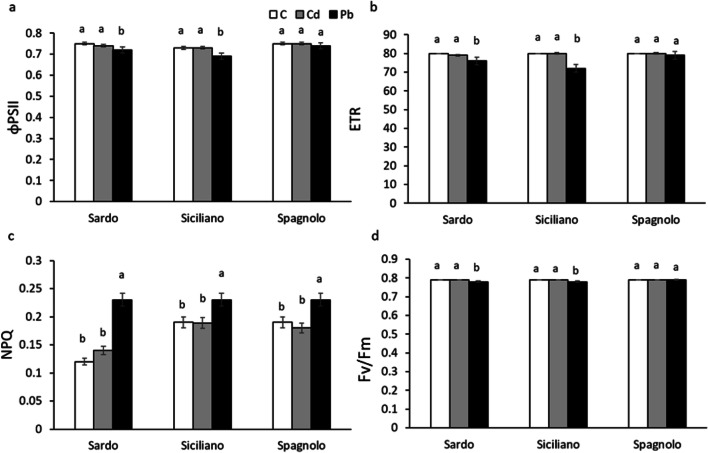
Quantum yield of PSII electron transport (ΦPSII) (a), electron transport rate (ETR) (b) nonphotochemical quenching (NPQ) (c), PSII maximum quantum yield (*F*_*v*_/*F*_*m*_), measured on control (C), Cd-treated, and Pb-treated Sardo, Siciliano, and Spagnolo cultivar. Data are the mean±SD, *n*=5. Different letters indicate significant differences among the treatments, according to Tukey’s post hoc test (*p*<0.05)

Cd and Pb exert different effects on the diverse components of photosynthetic apparatus; Cd seems to be more deleterious than Pb on light-harvesting pigments, but photosystem efficiency is more affected by Pb, even if the outcomes depend on intrinsic sensitivity of the different cardoon cultivars. The chlorophyll *a* fluorescence emission analyses showed that the quantum yield of PSII electron transport (ϕPSII), the electron transport rate (ETR), and the maximum PSII photochemical efficiency (*F*_*v*_*/F*_*m*_) were negatively affected only by Pb, in Sardo and Siciliano cultivars. Conversely, no effect following both metal treatments was detected in Spagnolo, confirming the highest capability of this cultivar to face the metal stress (Fig. 5a–c). The nonphotochemical quenching (NPQ) significantly rose under Pb treatment, in all cultivars, compared to Cd and control, indicating that under Cd treatment, photosynthetic apparatus promoted the thermal dissipation of energy not utilized in photochemistry (Fig. 5d) (Arena et al. 2017a, b). The higher photochemical efficiency of Spagnolo, compared to Sardo and Siciliano, was in accordance with the highest net photosynthetic rates measured in this cultivar under Cd and Pb treatments. From our data, it is evident that for the cultivars Sardo and Siciliano, the gas exchanges were more responsive than PSII photochemistry to metal stress, because the treatment with both Cd and Pb determined a significant reduction of net photosynthesis, together with a decline of stomatal number and photosynthetic pigment content. We suggest that the higher resistance of Spagnolo cultivar to Cd and Pb depends on an elevated capability to harvest and convert light in photochemistry, also in addition to a strong stomatal regulation allowing plants to carry out a fine-tuning of light and dark processes of photosynthesis.

### Antioxidant response

The three cultivars showed a different antioxidant power both in control plants and in response to metal injuries (Fig. 2s). It has been demonstrated that in natural ecosystems, the plant antioxidant production depends on several factors such as climate, soil, geographic location where plants grow, and, of course, species (George et al. 2004; Lebasky and Sharifi Ashoorabadi 2001; Ogaya et al. 2011); this could explain the different antioxidant power observed in the three cultivars also in the control condition. Particularly, the significantly lower value (*p*<0.01) of antioxidant capacity in Spagnolo control plants compared to Sardo and Siciliano could indicate a reduced need to develop a strong antioxidant system in this cultivar due to its intrinsic capacity to withstand metal stress.

Whereas in Sardo, no variation in antioxidant capacity among control and treated plants was found; in Siciliano Cd- and Pb-treated plants, this parameter significantly decreased compared to control, indicating a negligible role of the antioxidants under heavy metal stress in this cultivar (Fig. 2s). In addition, the Siciliano cultivar, although the higher constitutive antioxidant capacity, is not able to face the injuries under metal stress, as demonstrated by the sporadic occurrence of damaged stomata (Fig. 1s) and chloroplasts (Sorrentino et al. 2018).

Conversely, compared to control, Spagnolo cultivar showed a significant increase of antioxidant capacity in Cd- and even more in Pb-treated plants (Fig. 2s), suggesting a remarkable contribution of antioxidant compounds in plant defense against Cd and Pb injuries. Our results indicate that heavy metal stress influences antioxidant status, providing three cultivar-specific responses. However, as the FRAP assay only measures nonenzymatic (reductants) antioxidants in plant samples, we cannot exclude the occurrence of other effects involving specific enzymatic pathways, such as superoxide dismutase (SOD) or catalase (CAT) (Gjorgieva et al. 2013).

## Conclusions

This work clarifies several strategies of cardoon plants to cope with metal stress. Primarily, the modulation of the number and size of the stomata is proposed as a proxy for evaluating the tolerance to metals by potential phytoremediation plants. In fact, the increase of stomata of reduced size enhances gas exchange ability, determining the growth of healthy plants.

Some results indicate that *Cynara cardunculus* plants display and face metal injuries by active morphophysiological changes, regardless of the cultivar; specifically, root hair development and pigment content decrease. Other traits show instead a cultivar-specific behavior; particularly, photosynthetic pattern and stomata plasticity provide different responses, but only Spagnolo cardoon achieves a successful strategy preserving its morphophysiology. The stomata response follows two different patterns: the increase of the stomata area (i.e., Sardo and Siciliano) and the increase of the stomata number (i.e., Spagnolo). Only, this latter leads to a real resilience to metal stress, by improving the photosynthetic performance both in terms of CO_2_ absorption and light harvesting and conversion to photosystems. It is likely that the entrance of Cd and Pb into the cells, proved by the chemical analysis, induces a decrease in pigment concentration, which may be the first signal of an impending decline in net photosynthesis. This event, in turn, determines the onset of recovery mechanisms at the stomata level and adjustments in the photochemistry in Spagnolo cardoon. The increase of antioxidant capacity and the ability of Spagnolo plants to modify leaf structural and physiological traits under heavy metal contamination to maintain high photosynthetic efficiency should be considered an elective trait for its cultivation in polluted environments.

## Supplementary Information

ESM 1 (DOCX 268 kb)

ESM 2 (DOCX 130 kb)
