# Supplementary figures and images for: Facing metal stress by multiple strategies: morphophysiological responses of cardoon (Cynara cardunculus L.) grown in hydroponics
Source: Environ Sci Pollut Res Int. 2021 Mar 14;28(28):37616–26. doi: 10.1007/s11356-021-13242-9 (PMC8302550; doi:10.1007/s11356-021-13242-9)

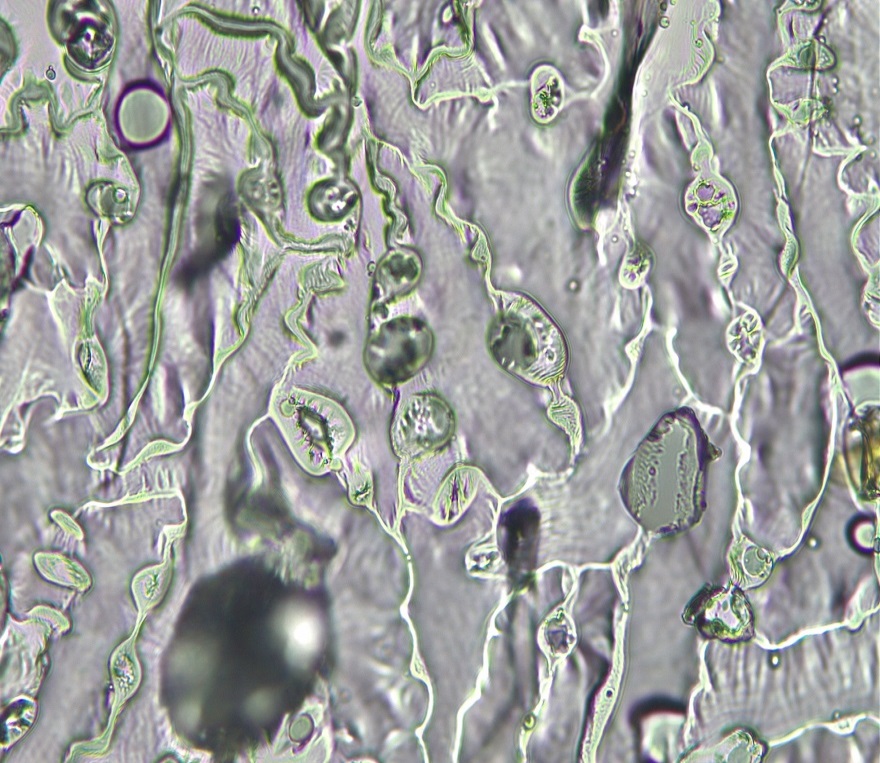


Figure 1S- Altered stomata observed in Cd-treated leaves of Siciliano cardoon

Supplement: Supplementary file 1 — (DOCX 268 kb) [file 11356_2021_13242_MOESM1_ESM.docx]
