# Supplementary material for: Facing metal stress by multiple strategies: morphophysiological responses of cardoon (Cynara cardunculus L.) grown in hydroponics
Source: Environ Sci Pollut Res Int. 2021 Mar 14;28(28):37616–26. doi: 10.1007/s11356-021-13242-9 (PMC8302550; doi:10.1007/s11356-021-13242-9)
Supplement: Supplementary file 2 — (DOCX 130 kb) [file 11356_2021_13242_MOESM2_ESM.docx]

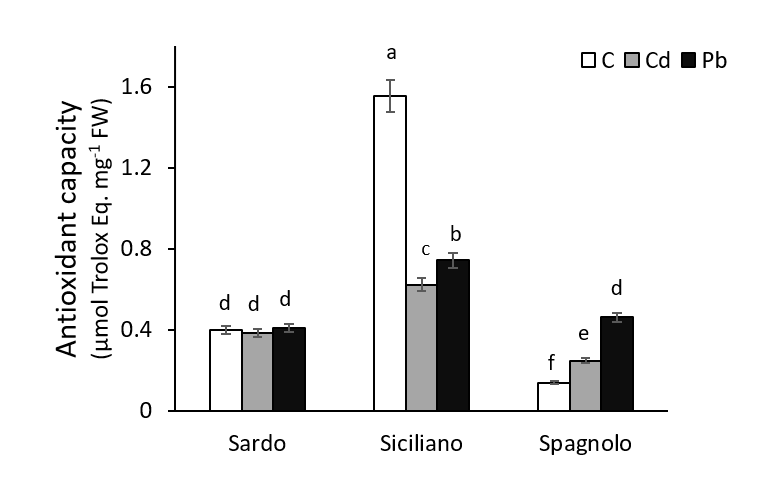


Figure 2s – Antioxidant capacity measured on control (C), Cd-treated and Pb-treated Sardo, Siciliano and Spagnolo cultivars. Data are mean±SD, n=3. Different letters indicate significant differences among the treatments, according to Tukey’s post-hoc test (p<0.05).
